# The earliest evidence of Acheulian occupation in Northwest Europe and the rediscovery of the *Moulin Quignon* site, Somme valley, France

**DOI:** 10.1038/s41598-019-49400-w

**Published:** 2019-09-11

**Authors:** Pierre Antoine, Marie-Hélène Moncel, Pierre Voinchet, Jean-Luc Locht, Daniel Amselem, David Hérisson, Arnaud Hurel, Jean-Jacques Bahain

**Affiliations:** 1 UMR 8591 Laboratoire de Géographie Physique, Environnements Quaternaires et actuels (CNRS- Univ. Paris 1- UPEC), 1 Place A. Briand, 92195 Meudon, Cedex France; 2 UMR 7194 Histoire naturelle de l’Homme préhistorique (MNHN- CNRS-UPVD), 1 rue René Panhard, 75013 Paris, France; 3 Institut National de la Recherche archéologique préventive (INRAP) Nord-Picardie, 32 avenue de l’Etoile du Sud, 80440 Glisy, France; 4 UMR 7041 ArScan (CNRS-Univ. Paris X Nanterre), Maison Archéologie & Ethnologie, René-Ginouvès, 21, allée de l’Université, 92023 Nanterre, Cedex France

**Keywords:** Archaeology, Palaeoclimate

## Abstract

The dispersal of hominin groups with an Acheulian technology and associated bifacial tools into northern latitudes is central to the debate over the timing of the oldest human occupation of Europe. New evidence resulting from the rediscovery and the dating of the historic site of *Moulin Quignon* demonstrates that the first Acheulian occupation north of 50°N occurred around 670–650 ka ago. The new archaeological assemblage was discovered in a sequence of fluvial sands and gravels overlying the chalk bedrock at a relative height of 40 m above the present-day maximal incision of the Somme River and dated by ESR on quartz to early MIS 16. More than 260 flint artefacts were recovered, including large flakes, cores and five bifaces. This discovery pushes back the age of the oldest Acheulian occupation of north-western Europe by more than 100 ka and bridges the gap between the archaeological records of northern France and England. It also challenges hominin dispersal models in Europe showing that hominins using bifacial technology, such as *Homo heidelbergensis*, were probably able to overcome cold climate conditions as early as 670–650 ka ago and reasserts the importance of the Somme valley, where Prehistory was born at the end of the 19^th^ century.

## Introduction

The dispersal of hominin groups with an Acheulian technology and associated bifacial tools towards northern latitudes is central to the debate over the timing of the oldest human occupation of Europe^1–7^. Indeed, while hominins occupied the South of Europe as early as 1.4 Ma ago^8^, their expansion into northern latitudes is thought to have occurred much later^1,9^. In the last decade, however, our understanding of the timing of the earliest occupation of north-western Europe has been updated. New discoveries have demonstrated that hominins occupied the southeast of England by the end of the Lower Pleistocene (≈800 ka), under cool continental conditions^1^. The earliest lithic assemblages with bifacial technology associated with Large Cutting Tools, cleavers and other heavy-duty tools, date to 1.75 Ma in East Africa^10,11^, 1.4 Ma in the Levant^12^, and close to 1 Ma in Spain^3,13,14^. Recently, occurrences of this Acheulian technology, which was originally defined in the Somme Valley^15^, have been dated to 700 ka in central France^4^ and between 610 and 670 ka in Italy^5^. In Northwest Europe however, and in particular in England and northern France (above 50°N), the earliest evidence of bifacial technology prior to this study was dated to MIS 14 around 550 ka^6,16^.

**Figure 1:**
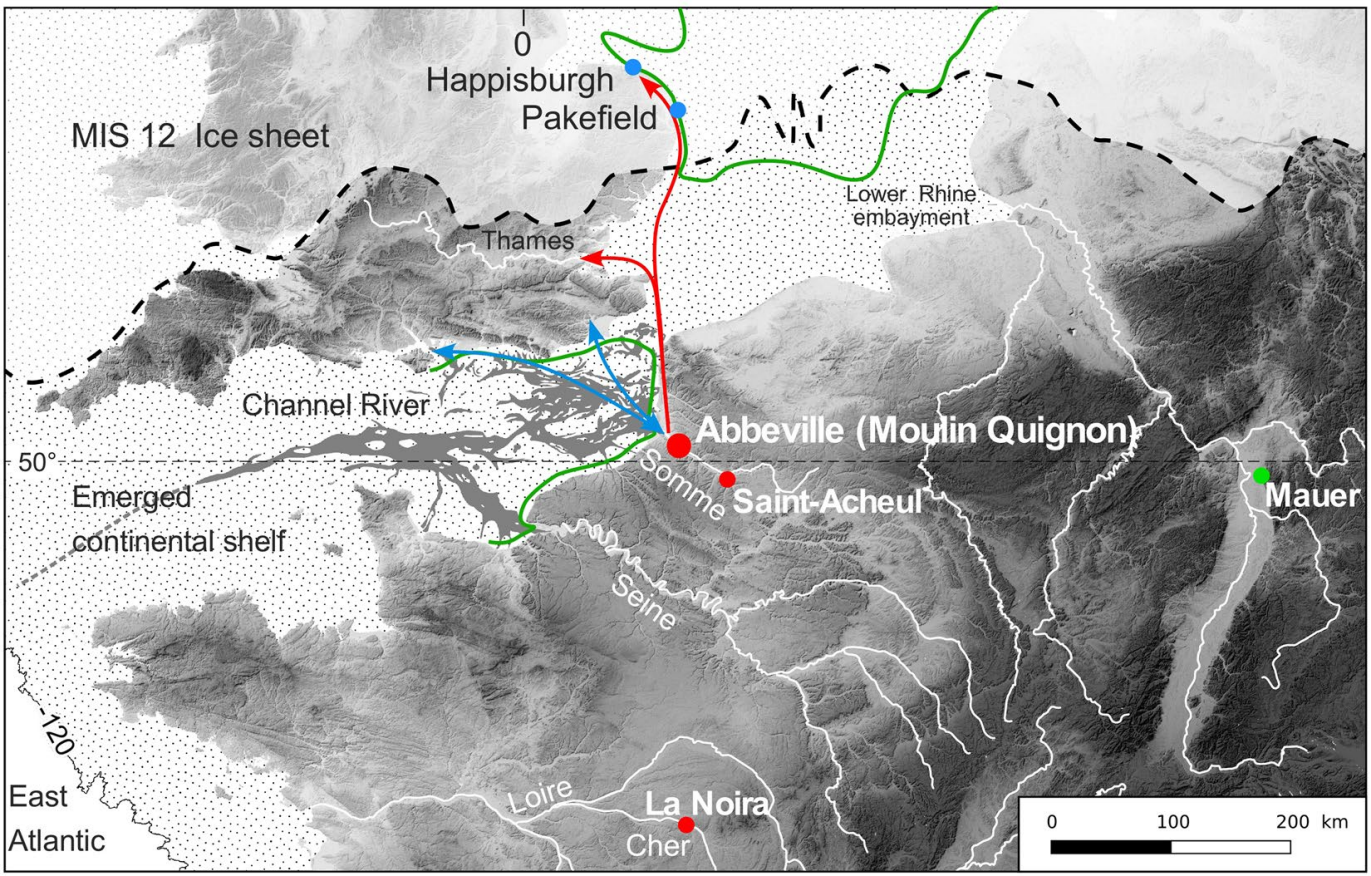
Location of Abbeville-Moulin Quignon and of some cores and flakes (blue dots), Acheulian sites (red dots), and of the *Homo heidelbergensis* type-site (Mauer) in Europe. MIS 12 ice sheet according to^21^, Cromerian shoreline (green) to^22^. Arrows: possible communication routes during interglacial (red), and glacial (blue) periods.

**Figure 2:**
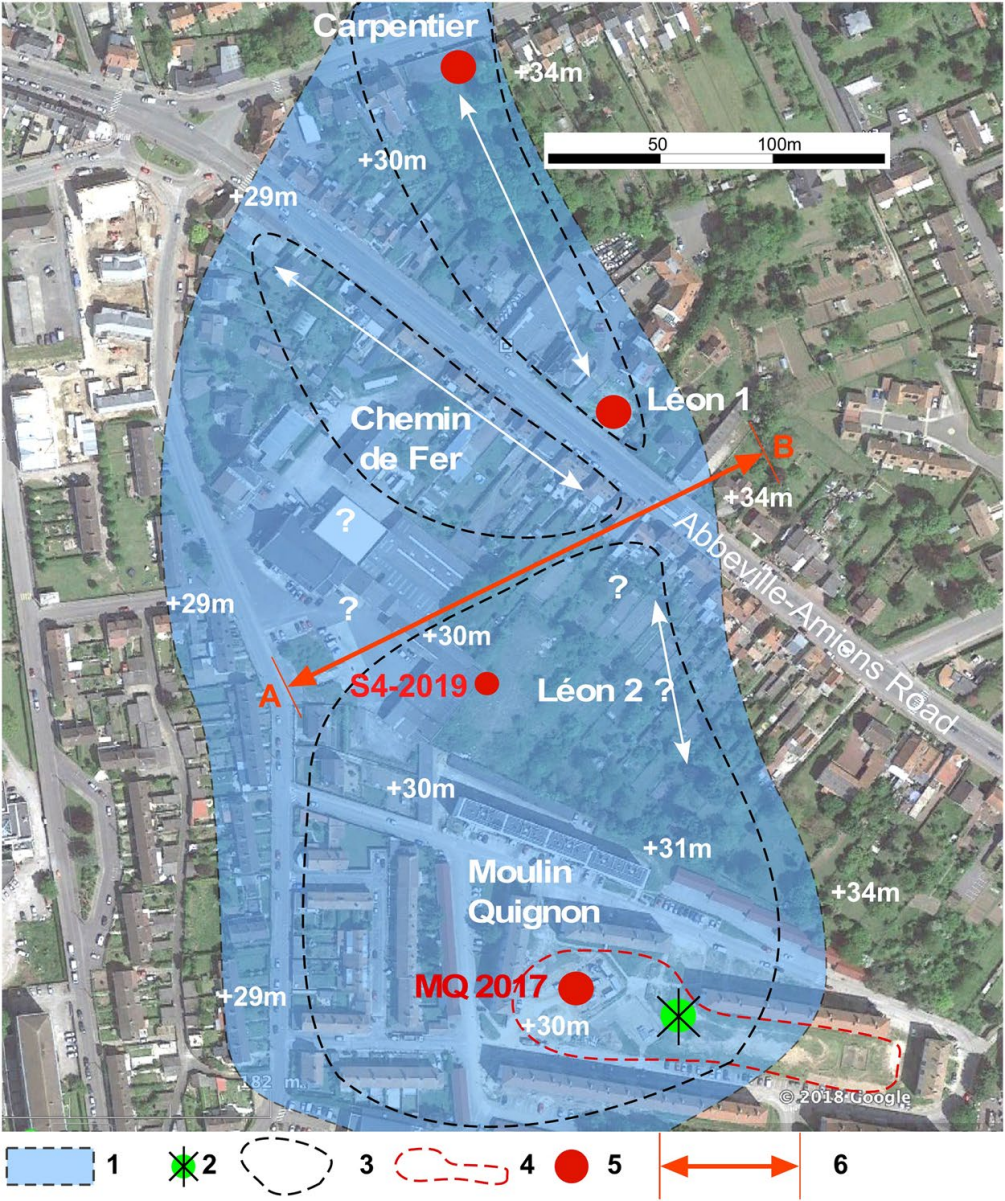
Detailed map of the Carpentier Alluvial Formation between Carpentier and Moulin Quignon (+40 m) with location of all the sites mentioned in this study according to recent^19,20,25^ and former^26–29^ data. Background image and topography according to Google Earth Pro 2018 (Image © 2018 DigitalGlobe), drawing by P. Antoine using Canvas Draw IV (https://www.canvasgfx.com/en/products/canvas-draw). (1) Reconstructed extension of Alluvial Formation VII: (Carpentier Formation, +40 m). (2) Location of the Moulin Quignon windmill at the end of the 19th Century. (3) Attempted reconstruction of the contour of the quarries at the end of the 19th Century according to^27,28^. (4) Area explored during the test-pit campaign in 2016 (17 test-pits). (5) Location of the recently described sequences (2012–2019). (6) (A,B): transect of Fig. 3 and Supplementary Fig. S5.

New discoveries at Abbeville and especially at the Moulin Quignon site (Somme Valley, Northern France, Figs 1 and 2), presented in this paper, drastically change the situation. Moulin Quignon (Fig. 2) is one of the sites where, between 1837 and 1868, the French archaeologist Jacques Boucher de Perthes discovered the first bifaces (“flint axes”) in fossil fluvial deposits^17^. This discovery led Charles Lyell to officially recognize “the great antiquity of Man” and was at the origin of the birth of Prehistory as a scientific field of enquiry^18^. A few years later (1863–1864), the discovery of human remains at Moulin Quignon, soon identified as a fraud perpetrated by quarry workers and recently dated by ^14^C as moderns^19^, damaged the reputation of the site which was subsequently forgotten for more than 150 years. However, the revision of the Moulin Quignon collections stored in the MNHN of Paris and including some bifaces and typical Cromerian fossil species demonstrated the crucial importance of this site for our knowledge of the earliest Acheulian of the Somme River basin and the Northwest of Europe^19,20^.

Here we report on the main discoveries made during new fieldwork undertaken at Moulin Quignon in 2017 following the rediscovery of the site during a test-pit campaign. We present geomorphic description and ESR-quartz dating of the fluvial deposits and a comparative analysis of the lithic industry.

## Geological Background: the Quaternary Fluvial Terraces of the Somme River Valley

During glacial periods^21^, the palaeo-Somme valley was one of the main tributaries of the Channel River, flowing into the Atlantic, and was a likely major route for human migrations between continental Europe and southern England^22^ (Fig. 1). The Somme River exhibits a stepped Quaternary fluvial terrace system incised into Upper Cretaceous chalk. Generally protected by a well-developed loess-palaeosol cover, this system is composed of a complex of 10 stepped alluvial formations spread between +5/6 m and +55 m above the maximal incision of the present-day valley in the Cretaceous chalk bedrock (Supplementary Figs S1, S2)^23^. The various bedrock steps and associated alluvial formations can be followed along at least a 50-km-long valley segment (Middle Somme) where they are separated by sharp incision banks of 5 to 6 m in height (Supplementary Fig. S1). In this very regular stepped fluvial system, accurate measurements of the relative altitude of the bedrock compared to the maximum incision of the valley are valuable data, characterizing the various alluvial formations. The relative height of the contact between the successive alluvial sequences and their respective bedrock steps thus can be used to securely insert the stratigraphic sequences of regional reference sites and associated archaeological layers as Moulin Quignon into the Somme River system^24^ (Supplementary Fig. S1). In addition, in the Somme valley, it has been shown using a multidisciplinary approach to the fluvial sequences (stratigraphy, sedimentology and palaeontology) that each alluvial formation corresponded to the morpho-sedimentary budget of a glacial-interglacial cycle^23,24^ (see detailed description in the caption of Supplementary Fig. S1). This approach to the system is strengthened by the combination of various geochronological data: ESR dating of fluvial quartz grains, ESR/U-series dating of large mammal teeth, TIMS dating on interglacial calcareous tufa and palaeomagnetic data for the oldest formations^23,24^. The Somme River terrace system represents a model of response to glacial-interglacial climatic cycles in a context of slow uplift on the edges of the Paris Basin (55 m for 1 Ma)^24^. Both the geochronological and palaeoenvironmental contexts of this system are presently among the most clearly established in Europe for the last 1 Ma^23–25^, allowing for an accurate control of the chronology of the archaeological data and especially of those resulting from the new discoveries made at Moulin Quignon.

## Results

### Stratigraphy and correlations with the Carpentier reference sequence

**Figure 3:**
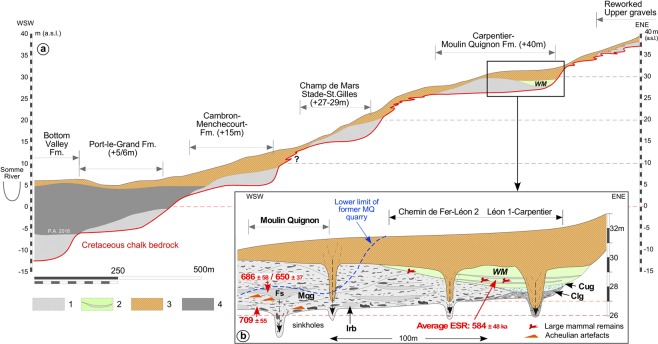
Cross-section of the terrace system of the right bank of the Somme River at Abbeville. (**a**) General profile. (**b**) Detailed stratigraphic relations between Moulin Quignon and Carpentier sites on the +40 m alluvial formation and ESR-quartz dating results (see: Fig. 2 and Supplementary Fig. S5 for location of the various observation points). The geometry of the Quaternary deposits and of the chalk bedrock relies on data originating from the present work for Moulin Quignon, on^22^, for the Carpentier and Léon quarries, completed by the diagrams and data from (26 to 29). This figure clearly illustrates the stratigraphic relationships between the sequences of Carrière Carpentier and Moulin Quignon. (1) Periglacial fluvial gravels, sands and sandy silts (main unit of the various terrace bodies). (2) Carpentier Marne blanche/White marl (WM) complex: interglacial calcareous silts and sandy silts with oncolith sand layers and large mammal remains (Cromerian III/MIS 15). (3) Undifferentiated slope deposits: reworked gravels and palaeosols. (4) Fluvial organic silts, peat and estuarine sands (Holocene infilling). WM: White marl. Cug: Carpentier Upper gravels: well-sorted medium flint gravels with calcareous sandy matrix, interstratified calcareous silt lenses and large mammal remains. Clg: Carpentier Lower Gravels: poorly sorted and heterometric flint gravels including numerous unrolled flint nodules and chalk blocks packed in a calcareous sandy matrix. Mqg: Non-calcareous, coarse, poorly stratified sandy gravels with iron and manganese oxide coatings at the base. Irb: large rounded blocks of reworked Tertiary sandstone (ice-rafted). Fs: Lens of laminated non-calcareous fluvial sands. Average ESR dating of the WM according to^25^.

During the revision of the Quaternary Somme terrace sequences at Abbeville^25^, new fieldwork was first conducted in the Carpentier and Léon 1 quarries (Fig. 2, Supplementary Fig. S3), well known for their rich paleontological assemblages^26–29^. These two neighbouring sites exhibit fluvial sequences overlying the same bedrock step (+27 m a.s.l.), located at a relative height of +40 m in relation to the maximal incision of the present-day valley (Alluvial Formation VII, Fig. 3a, Supplementary Fig. S2). Both the age and the interglacial characteristics of the calcareous sandy-silts of the *Marne blanche* or *White marl* capping the fluvial sequence of the Carpentier Formation have been firmly established^25^ using a reliable combination of: (i) ESR (quartz) and ESR/U-series (large mammal tooth) dating, (ii) large and micro mammal and mollusc assemblages, (iii) the occurrence of oncolith layers (algal balls) resulting from cyano-bacterial activity under full temperate conditions, and (iv) finally, the insertion of the Carpentier Formation within the Somme valley Quaternary terrace system (Formation VII in a system where Formation X is dated to about 0.9–1 Ma and formation I is dated to the Late Saalian-Eemian glacial-interglacial cycle). The rich large mammal faunal assemblage recovered from the *White marl* at Carpentier quarry clearly characterizes a temperate interglacial period and can be biostratigraphically correlated to a Late Cromerian interglacial^25^. Combined with the geochronological data obtained by both ESR-quartz (n = 6) from the sandy lenses included in the fluvial deposit of the *White marl* and ESR/U-series on cervid teeth (n = 2) (mean age of the whole set of data: 584 ± 48 ka), this interglacial stage can be allocated to the Cromerian III Interglacial of the formal north-western European stratigraphy and correlated with MIS 15^30^. The *White marl* from Carpentier is thus one of the best-dated series from the Cromerian III interglacial in Western European river systems and represents a fundamental cornerstone for the interpretation and dating of the Moulin Quignon sequence and more generally for the Quaternary terrace system of the Somme River.

In this context, thanks to the renovation of the suburb of Abbeville, new investigations were undertaken in 2016–2017 at Moulin Quignon (50°06′18″N/1°50′89″E, Figs 1 and 2), leading, 170 years later, to the rediscovery of this emblematic Palaeolithic site (Supplementary Fig. S4). In 2016, seventeen 4-to-5-m-deep test-pits were excavated, resulting in the discovery of undisturbed, fluvial gravels and sands, well-preserved below thick layers of reworked sandy gravels (from the former quarry) and modern dump deposits. The average bedrock altitude measured at Moulin Quignon (26.5 m a.s.l.) in both test pits and in the archaeological excavation is very close to the altitudes obtained for the Léon 1 and Carpentier quarries, located 200 and 400 m north of the site, respectively (Figs 2 and 3). This approach demonstrates that the three alluvial sequences are located on the same bedrock step (Fig. 2, Supplementary Fig. S5). Consequently, compared to the maximal incision of the valley at Abbeville below the alluvial plain deposits (≈−12 m below sea level), the relative elevation of the Moulin Quignon alluvial formation (+40 m) allows for its attribution to Alluvial Formation VII of the Somme system (Supplementary Figs S1, S2).

In addition, we compiled all the available stratigraphic information from Alluvial Formation VII, from the area located between Carpentier and Moulin Quignon, taking in account recent observations at Carpentier, Léon 1 and Moulin Quignon (Fig. 2) made between 2012 and 2017, and all the former data available from D’Ault-du-Mesnil^26^, Commont^27^ and Breuil^28,29^.

This approach was completed by a new test-pit campaign during spring 2019 over an abandoned area further to the north (140 to 180 m) of the Moulin Quignon 2017 excavation, closer to the Carpentier Quarry (Fig. 2). This extensive survey (17 test pits on 2 600 m^2^) allowed the discovery of *in situ* remnants of fluvial gravels and sands (thickness about 1 m) overlaying the same chalk bedrock step than in Carpentier Quarry and Moulin Quignon 2017 excavation. Moreover, in one of the test-pits (S4-2019, Fig. 2), ten Acheulean artefacts were discovered within the sandy gravels demonstrating the extension of the Moulin Quignon Acheulean site over more than 150 m to the North. These results strongly support the direct stratigraphic correlation between the fluvial sequences from Moulin Quignon and Carrière Carpentier.

The synthesis illustrated by Supplementary Figs S5 and S6 shows that the Carpentier interglacial deposit, the *White marl*, extends below the road towards the area corresponding to the Chemin de Fer and Léon 2 quarries located at less than 100 m from the area explored at Moulin Quignon in 2016–2017 (Fig. 2, Supplementary. Fig. S5). It is clear from this that the interglacial calcareous facies of the *White marl* is only preserved in sequences located in the external part of the alluvial formation. This configuration results from the occurrence of a lateral channel close to the north-east bank of the Palaeo-Somme valley, whereas interglacial deposits are not preserved towards the central part of the former river valley, showing a markedly thicker lower gravel unit (≈3 to 3.5 m), like in the Moulin Quignon area (Fig. 3). Thus, combining the new observations and former data, we can demonstrate: i) that all the fluvial sequences from the area reported from more than 150 years overlie the same bedrock step incised by the Somme River between 26 and 27 m a.s.l. (relative height of 39 to 40 m), and ii) that a clear distinction can be made in all the sections reported by former authors^26–29,31^, between the fluvial gravels, systematically located below the interglacial deposit of the *White marl*, and the slope deposit sequence made up of heterogeneous layers of reddish clayey gravels, clayey sands, sandy loess and palaeosols, which always appear above the *White marl* (Fig. 3b, Supplementary Fig. S5). Given the synthesis of the stratigraphic information exposed above, the geochronological data from the deposits of the Carpentier quarry, and especially the ESR and ESR/U-series dates from the interglacial deposits of the *White marl*, can be used to reliably infer the age of the Moulin Quignon findings.

### Electron spin resonance (ESR) dating

**Figure 4:**
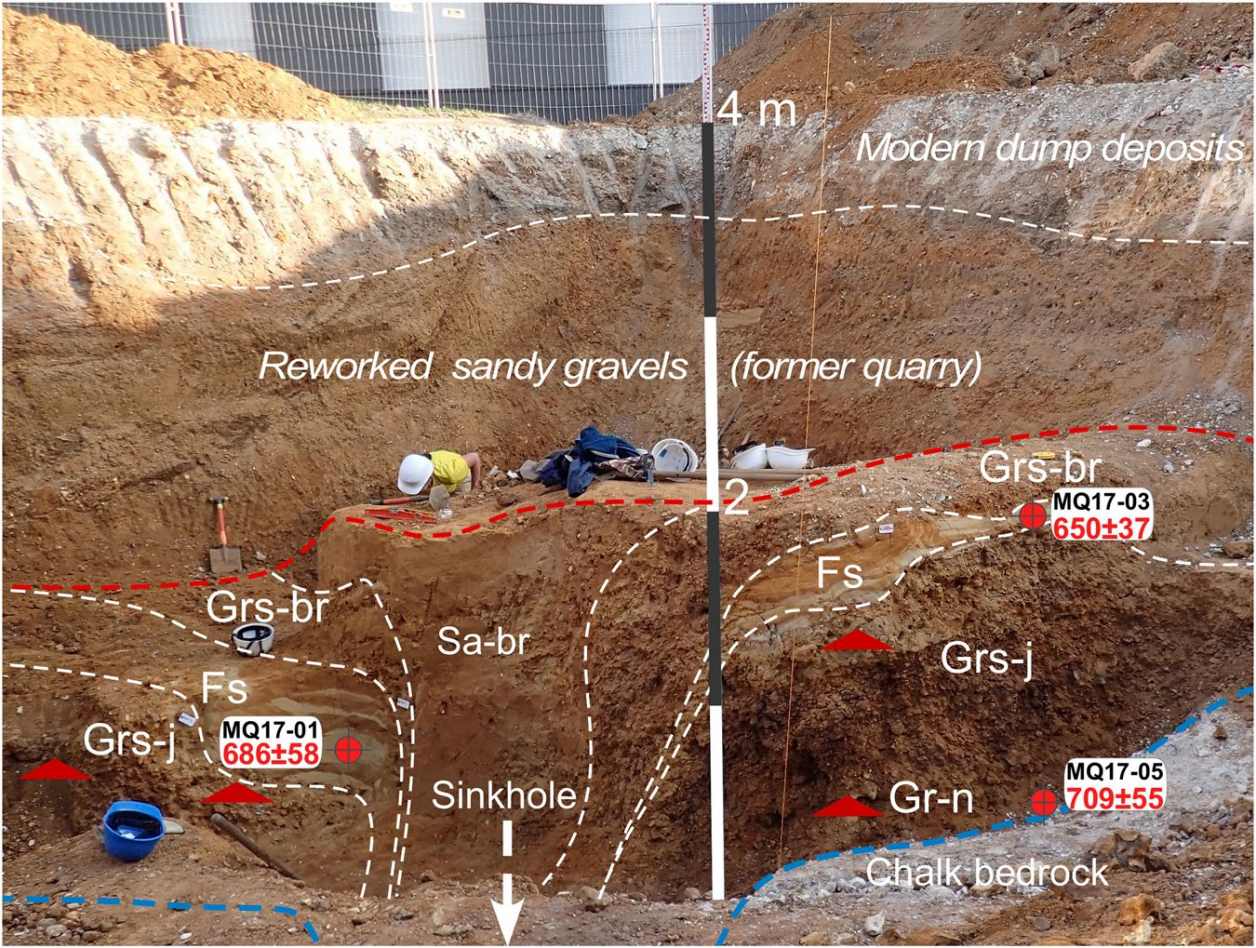
Stratigraphic sequence exposed during the excavation (height: 4 m). From the top below the reworked sandy gravels from the former Moulin Quignon quarry: Sa-br: Brown-red compact clayey sands preserved in deep dissolution sinkholes. Grs-br: brownish to reddish sandy heterometric clayey rounded gravels with small (reworked) tertiary pebbles. Fs: Yellow fine to medium laminated sands with thick (2–5 cm) red to orange oxidized bands. At its base, in places this unit shows a layer of laminated greyish clayey silts with thin oxidized orange (Fe) and black (FeMn) bands. Grs-j: Heterometric rounded sandy flint gravels with abundant yellow sandy matrix and irregular reddish to orange oxidation (Fe) bands and with (reworked) tertiary pebbles (1–4 cm). Gr-n: Strongly heterometric rounded flint gravels, without any matrix, (mainly 2–4 cm but including irregular elongated nodules up to 30 cm) and scattered (reworked) tertiary pebbles (1–4 cm). This facies is characterized by strong weathering indicated by the occurrence of brownish to blackish Fe-Mn coatings on all the flint nodules and pebbles. All the units described above are free of CaCO_3_ due to dissolution and weathering processes that affected the whole sequence after its deposition by the river. Chalk: weathered (soft) Upper Cretaceous chalk bedrock including numerous irregular large elongated flint nodules (20–40 cm in length).

Three samples of sandy sediments were extracted during the 2017 archaeological excavation from the main stratigraphic units and *in situ* gamma-ray measurements were performed in each sampling hole (Fig. 4). Details of the Electron Spin Resonance (ESR) dating method applied on sedimentary quartz are given in the Method part.

The results obtained for the three samples from Moulin Quignon, using the aluminium (Al) and lithium titanium (Ti-Li) centres of quartz, are consistent with each other (overlapping of uncertainty domains at 2 σ) (Supplementary Table S1). This consistency makes it possible to combine the results and calculate a weighted mean age for each sample using the Isoplot 3.0 software^32^. For samples MQ17-01 and MQ17-03, from the fluvial sand layer (Fs) overlying the main gravel body (Grs-j), the results are respectively 686 ± 58 ka and 650 ± 37 ka (Table 1). Similarly, an average age of 672 ± 54 ka was obtained for the whole fluvial sands and gravels formation of Moulin Quignon (Table 1), confirming the great antiquity of the site.

**Table 1:** Annual doses, equivalent doses and ages of the Moulin Quignon fluvial sediments obtained using Al and Ti-Li centres and mean ages determined for each sample and the fluvial formation.

Samples	Units	D_a_ (µGy/a)	D_e_ (Gy)	Ages (ka)	Mean Ages (ka)	Mean Age (ka)
MQ17-01 Al	Fs	**505 **±** 20**	343 ± 36	**679 **±** 74**	686 ± 58	**672 ± 54**
MQ17-01 Ti-Li	Fs		348 ± 25	**689 **±** 94**		
MQ17-03 Al	Fs	**661 **±** 22**	447 ± 78	**677** ± **61**	650 ± 37	
MQ17-03 Ti-Li	Fs		418 ± 105	**633** ± **48**		
MQ17-05 Al	Gr-n (base)	**346** ± **15**	257 ± 86	**742 **±** 89**	709 ± 55	
MQ17-05 Ti-Li	Gr-n (base)		239 ± 100	**689 **±** 71**		

Age results are given with 2σ uncertainty. Mean ages and associated uncertainties were determined using Isoplot 3.0 software^32^.

### Palaeolithic artefacts

**Figure 5:**
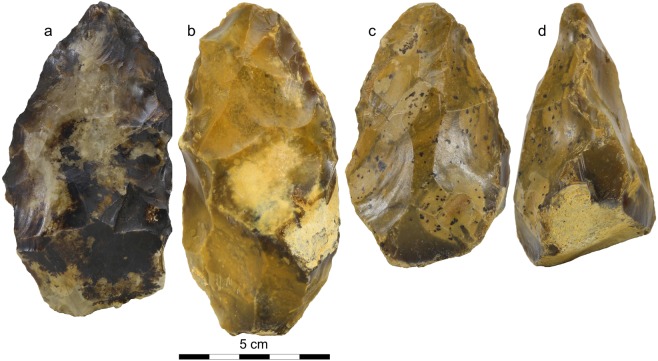
Photographs of three of the bifaces discovered during the archaeological excavation at Moulin Quignon. (**a**) Biface with extended black coatings of Fe-Mn oxides typical of lithic artefacts discovered in the lower part of the Moulin Quignon sequence (GR-n layer: see Fig. 4 and Supplementary Fig. S6). (**b**) Biface from Grs-j unit. (**c,d**) facial and lateral view of a biface from Grs-j unit with thick base.

Unexpectedly, typical Palaeolithic artefacts including flakes, flake-tools, cores and one biface were discovered at depths ranging between 3.5 to 4 m in two of the test pits, in the sandy gravels of the terrace deposits preserved by former quarry works. The taphonomy of this archaeological assemblage is described later in Discussion section. In 2017, an archaeological excavation located around the two positive test pits (Fig. 4, Supplementary Fig. S4) yielded several additional new artefacts. With a total of 244 flakes, 13 cores and 5 bifaces (Fig. 5) discovered *in situ* in the lower part of the alluvial deposits, the excavation provided us with a lithic assemblage large enough to carry out a full technological analysis. The taphonomy of this archaeological assemblage is described later in the Discussion section.

The lithic series are made from local flint nodules available both in the alluvial gravels and in the chalk bedrock. The flakes belong to a complete reduction process including mainly large flakes (mostly between 40 and 80 mm long). The cortical flakes (first phases) indicate the use of oval or irregular flint nodules. Unipolar or unipolar convergent removals eliminated the cortex by series of thick flakes. The platform was prepared or remains cortical. The majority of the flakes have a cortical back and for some of them both a cortical back and butt. This attests the use of the nodule sides or core edges for eliminating the cortex and starting the flaking process. Flakes without cortex show that the debitage continued by unipolar, centripetal and/or crossed removals. A frequent back suggests the recurrent use of the core edges as a guide for this debitage. Flakes are thick, some truncating a large part of the cores. The flake cross-sections indicate flaking surfaces with angular facets and deep scars. Some elongated flakes (laminar flakes) are mainly due to the use of prominent scar ridges or the core edges. Butts are plain, but also punctiform, dihedral and facetted. Few flakes are hinged, indicating good general management of the debitage angles. The main characteristic of thick and large butts is an open angle (50–60°). Impact points were regularly located far from the core edges. There are six retouched pieces and a further 30 pieces with possible retouch. They are an end-scraper on a large and elongated cortical flake (Supplementary Fig. S7), two convergent tools (point) and some retouches on a long edge.

The cores indicate either crude and opportunistic flaking, or unipolar/centripetal debitage on one, two (orthogonal or bifacial) or multiple surfaces. Cores are exhausted or still in progress. Scar size indicates a large variety of end-product size and shape. Some cores are crudely flaked, residues of extremities of nodules with one flaking surface, few scars and an occasional prepared platform. When the cortical area is suitable, the platform is not prepared. Others are unifacial cores with unipolar, centripetal and crossed removals more or less covering the surfaces, with a partial prepared platform. Some hinged removals attest poor flaking management for some cores. The orthogonal cores and the bifacial cores sometimes indicate a degree of independence from the raw material by series of removals with no link with nodule shape and thus limited blank constraints. We can mention in particular two cores with peripheral unipolar removals with a prepared platform (semi-rotating-like core). The biggest core (155 mm long) shows several flaking surfaces around part of the periphery and on one large surface by centripetal, invasive and deep removals (Supplementary Fig. S7). The core was turned in the hands according to the suitable angles and re-created some angles.

A few flakes can be related to bifacial technology. They are completed by five bifaces which show wide diversity in shape and shaping mode (Supplementary Fig. S8):

- Crudely worked biface made by large and deep removals by alternate shaping on both faces with a tip modified by a small transversal removal (use?). The cross-section is asymmetrical and similar to the shape of the nodule. Retouches and crushing marks are visible on the edges.
- Triangular elongated biface without cortex. It is asymmetrical (lateral back created by abrupt removals), made by large, crossed and flat removals. Special attention was paid to the upper part of the tool with small removals and retouches. We can mention an invasive removal from the base covering the lower part on one face.
- Triangular and symmetrical biface with a cortical and thick base. Shaping by alternate large and deep removals manages the complete bifacial volume. Special attention was paid to the tip, modified by several removals, and retouch is observed on the whole periphery of the tool. The edges remain sinuous.
- Oval and symmetrical biface with cortical patches on one face and a back. Centripetal removals globally shape the artefact with careful attention to the oval and thin extremity. Retouches are visible everywhere.
- Broken and asymmetrical biface made by large and convergent removals on one face, the fracture showing flexion. Transversal removals manage the base. Final and deep unifacial or bifacial retouches cover the edges. Some crushing attests to percussion on hard material.

## Discussion

The fluvial deposits preserved at Moulin Quignon show a homogeneous stratigraphic succession throughout the whole surveyed area (Fig. 3b, Supplementary Fig. 5). The various facies observed in the alluvial sequence (Fig. 4) and their organization clearly present the same characteristics compared to fluvial deposits from the lower units (coarse gravels) of the alluvial sequences of the Quaternary terraces of the Somme basin^23,24^ and exhibit the characteristics of braided river channels^33^. The interpretation that the Moulin Quignon gravels and sands were deposited in a periglacial environment is first based on the occurrence, at the base and within the fluvial gravels, of some very large rounded Tertiary sandstone blocks (up to 0.5 to 0.8 m in length), reworked during episodes of periglacial mudflows from the slopes of the valley towards the alluvial plain (Fig. 3b, Irb, Supplementary Fig. S6). Indeed, in the alluvial formations of the Somme River (as in numerous rivers of Northwest Europe), these large ice-rafted blocks are described within, or at the very base, of Quaternary alluvial formations^24^. The reworking of these sometime huge rounded blocks (>1 ton) from the slopes to the alluvial plains, and their subsequent transport by fluvial systems, imply that major episodes of solifluction occurred on the slopes, resulting from multiple thawing episodes of the upper permafrost layer (active layer) and that extremely strong river dynamics transported large ice-rafted blocks and heavy loads of coarse and poorly sorted material (flint nodules and chalk blocks) during spring break-ups and floods. These processes characterize the full glacial conditions that occurred several times during Quaternary glacial periods in Northwest Europe^34^ and are typical of the braided river systems in Quaternary periglacial and present-day northern tundra environments^35,36^. The deposition of the Moulin Quignon sequence during periglacial conditions is also shown by: (i) the occurrence of well-sorted flint gravel layers including a main stratified sand lens (Fig. 4, Fs) deposited in a shallow channel structure (at least 5 to 6 m wide) characterising braided river systems, (ii) a very low proportion of sandy matrix as well as iron oxide and manganese coatings on the gravels (Fig. 4, Gr-n) that are both typical of the basal units of the fluvial sequences of the other Somme terraces^37^ and, (iii) the occurrence of numerous large and poorly rolled flint blocks (20 to 40 cm in length at the base of Gr-n), as generally found at the base of the gravel bars and islands in braided river systems (lag deposits).

Finer facies (grey silty sands, yellow laminated sands) observed in Moulin Quignon sections may reflect different climatic conditions, but no bio-indicators, which would enable us to refine the palaeoenvironmental interpretation, are preserved due to post-depositional weathering processes. The occurrence of pollen has been tested without success in the thin layers of clayey-silty sands occurring at the base of the main sandy lens (Fs in Figs 3 and 4). These fine-grained sediments could very well have been deposited in the same braided river environment and trapped in abandoned shallow channels occurring between gravel bars at the end of a flood episode.

Finally, the gravels at Moulin Quignon exhibit a totally different set of features from those of the slope deposits covering the *White marl* at Carpentier (as well as at Léon 1 and in other formerly excavated quarries). These very heterogeneous, badly sorted, and deformed deposits, including clayey gravely deposits, are clearly not fluvial and conversely, result from multiple episodes of reworking by hillwash and periglacial slope processes of older alluvial formations, as well as former clayey palaeosol horizons and clay with flints located higher on the slopes (Fig. 3, No. 3 Supplementary Figs 5, 6).

In addition, the facies and stratigraphic succession observed in the new Moulin Quignon excavation are very similar to the descriptions published in the 19^th^ century by Boucher de Perthes^17^ and Prestwich^38^, in terms of the overall thickness of the sequences and sedimentary facies. Boucher de Perthes described a sequence composed of gravel beds and sand layers, with a thickness (±4.5 m) perfectly compatible with the difference between the altitude of the bedrock step measured during the new excavations and test pits (26.5 m) and that of the surface topography (30.5 to 31 m). Moreover, at the very base of the fluvial sequence, we also found a layer of gravels strongly coloured by iron and manganese oxides (Fig. 4, Gr-n), which was described by Boucher de Perthes (unit 5 “Couche noire”) and Prestwich (*the Black band*) in the same stratigraphic position^17,38^, and in which we also discovered Palaeolithic artefacts.

At Moulin Quignon a strong post-depositional weathering of the gravels is attested by the formation of a black clayey layer, very rich in iron and manganese oxides at its base in contact with the chalky substratum (Bβ horizon). In the lower 30 to 50 cm of all the sections, a strong accumulation of oxides forming coatings on the surface of flint nodules and pebbles, already noted by former authors^17,38^, indicates marked post-sedimentary weathering of the sequences. This process is accentuated by the position of the site on the west-facing slope of the Somme valley, where the thick calcareous loess cover, which protects the alluvial sequences from decalcification elsewhere, is very thin or absent^25^. The absence of a protective carbonated loess cover strongly enhanced the formation of deep dissolution sinkholes in the chalk bedrock (Figs 3 and 4). These sinkholes (3–4 m in depth/1–2 m in diameter) were described during the 19^th^ century at Moulin Quignon, and in the Carpentier and Léon quarries^26–28^. However, they are of limited diameter and can thus be easily avoided when measuring the altitude of the bedrock of the alluvial sequence. In contrast, thick and permeable carbonated loess beds (several meters), such as those occurring at the top of the fluvial sequences on the left bank of the Somme River (CaCO_3_: ≈15%), protect underlying layers (loess or/and fluvial) from dissolution by meteoritic water. In these configurations, the basal contact between the alluvial sequences and the chalk bedrock shows no solution sinkholes^23,24^.

The detailed extension of the Carpentier Alluvial Formation on the +40 m bedrock step, illustrated in Supplementary Fig. S5 and Fig. 2, thus shows that the Moulin Quignon periglacial gravel and sand formation corresponds to the lateral extension to the south of the Carpentier Alluvial Formation (Figs 2 and 3b).

From a chronological point of view, the previously established connection between the Carpentier, Léon 1 & 2, Chemin de Fer and Moulin Quignon sequences enables us to: i) correlate the Moulin Quignon sequence with the sequence at Carpentier, which contains a paleontological assemblage related to the Cromerian III interglacial; and ii) use the well-established ESR and ESR/U-series chronology of the *White marl* of the Carpentier quarry to infer a reliable age for the Moulin Quignon fluvial deposits. As a result, we are able to date the fluvial deposits containing archaeological material at Moulin Quignon to the cold stage (glacial) immediately predating the Cromerian interglacial III/MIS 15, i.e., Cromerian Glacial B or MIS 16^30,39^.

**Figure 6:**
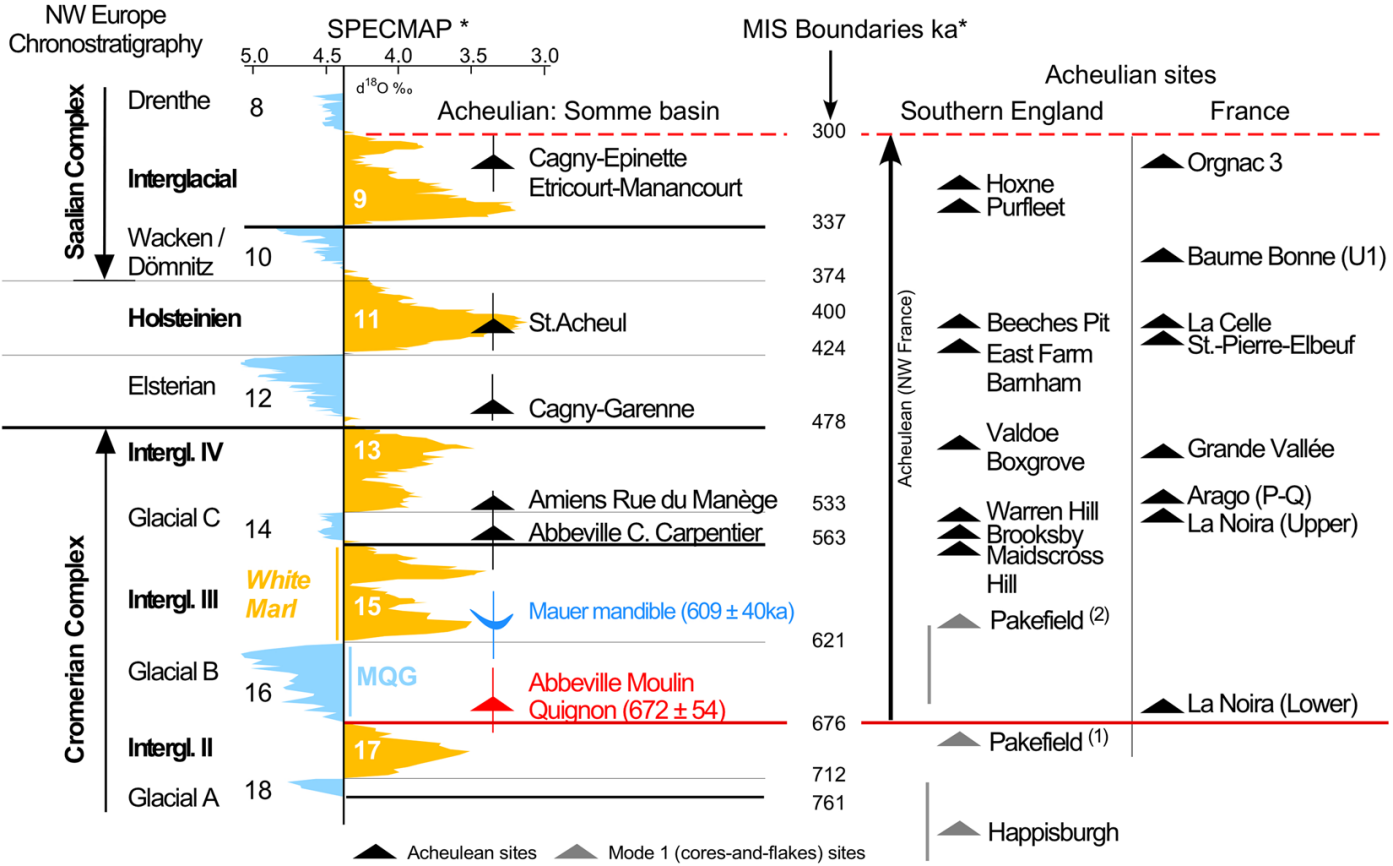
Chronological location of Moulin Quignon site with respect to the north-western Europe Acheulian and core-and-flake sites, modified from^25^. MIS chronology according to^39^, Northwest European formal stratigraphy according to^30^. Age of Pakefield site: (1) according to^9^ and (2) according to^6^.

This chronostratigraphic interpretation is supported by the ESR-quartz ages obtained from the three samples from the Moulin Quignon fluvial sequence (average: 672 ± 54 ka). The age of the Acheulian artefacts discovered at Moulin Quignon within the fluvial deposits (Fig. 5) is thus probably in the range of about 670 to 650 ka (Fig. 6). An older age would be very unlikely because: (i) the artefacts were recovered from a cold period deposit that thus cannot be allocated to an interglacial period such as MIS 17 interglacial whose terminated at 676 ka^39^. In addition, the homogeneous character of the physical characteristics (abrasion and patina) of the archaeological pieces from Moulin Quignon exclude reworking from an older terrace formation and no traces of occupation have ever been described in the older alluvial sequences of the Somme system (Formation VIII or Saveuse Fm., Supplementary Figs S1, S2) despite more than 150 years of intensive research in the area. We are therefore clearly faced with artefacts that belong to the earliest known Acheulian occupation ever dated in the Somme basin and, more generally, in north-western Europe.

Most of the Acheulian artefacts were discovered in the sandy flint gravels at a depth ranging between 3 and 3.5 m (Fig. 4, Grs-j). Only two bifaces and a few flakes showing a thick black patina occur at the very base of the fluvial sequence (Fig. 4, Gr-n, Fig. 5). The flint artefacts (Fig. 5, Supplementary Figs S7, S8) generally show fairly clear edge abrasion reflecting transport in gravely to sandy-gravely sedimentary deposits during highly dynamic fluvial events. However, a significant proportion of artefacts show weakly abraded edges. The archaeological excavation also shows that the artefacts are not regularly distributed in the fluvial deposit but rather, are localized in spatially discrete concentrations separated by archaeologically sterile zones. The Acheulian artefacts discovered at Moulin Quignon probably represent human occupations during a cold period, located in the former alluvial plain of the Somme River on a gravel bar at a short distance from the regularly submerged valley slope during spring floods. During these floods, the flint artefacts were displaced over short distances (a few meters), before being buried in the sand-gravely material. However, it is highly likely that human occupations from this period only correspond to short incursions of human groups during the summer season.

The flakes from Moulin Quignon are technologically similar to those from Happisburgh (900 ka) and Pakefield (700 ka) in England^1,9^, described as Mode 1-type (cores-and-flakes) lithic industries, and to the artefacts from Amiens-Rue du Manège in the Somme Valley^16^. Scars on the flakes are mainly unipolar or centripetal with thick and large flat butts characterised by an open angle. These artefacts are the earliest evidence of bifacial technology in Northwest Europe. At Moulin Quignon, the presence of five bifaces (Fig. 5, Supplementary Fig. S8), in addition to flakes resulting from the bifacial shaping process, clearly attest to the presence of this technology at least 100 ka earlier than previously known in the North of France (Fig. 6). The bifaces, with diverse forms and modes of shaping, range from crudely made to extensively worked artefacts (Fig. 5, Supplementary Figs S7, S8) and exhibit similar characteristics to those found by Boucher-de-Perthes in 1837–1868^17^ and classified as “Abbevillian” in the past^40^. They are also similar to bifaces from other 700–650 ka old European series^2,4,5^. The new results from Moulin Quignon and the synthesis of the data concerning the early Acheulian lithic assemblages of northern France lead us to underline the diversity of bifaces produced in the same assemblage as well as a total mastery of bifacial technology, thus excluding the idea that a primeval cultural phase occurred first, especially in this part of France^2,41,42^.

## Conclusion

The rediscovery of the former Moulin Quignon site, the new archaeological findings and the ESR dating of the fluvial sequence contribute to bridging the gap between France and England and help us to revise our definition of the earliest occupation of Northwest Europe by *Homo heidelbergensis*^43,44^. Indeed, in the south of Europe, Italian and Spanish sites yield traces of early occupations between 1.4 and 1.2 Ma, despite critical reviews^45^, and Acheulian occupations older than 600 ka^3,5^. The first Acheulian evidence in the Centre of France is dated around 700 ka^4^ and no Acheulian bifaces are known in England before 500 ka^2^. Traditions characterized by an elaborate bifacial technology are now known to occur in north-western Europe at the same date (700–600 ka) as in southern Europe. The rediscovery and absolute dating of the Moulin Quignon site also contributes to our knowledge of the origin of the Acheulian and the timing of its dispersal in Europe. Our results confirm the age of the earliest well-controlled bifacial technology both in the North and South of Europe^2,4,5^. They also indicate that hominins associated with an Acheulian technology, in all likelihood *Homo heidelbergensis*, were able to expand into northern latitudes as early as 670–650 ka, which is much earlier than previously anticipated, and not only during interglacial periods.

## Methods

Field methods can be found in^25^. Here, we describe the Electron Spin Resonance (ESR) dating techniques used to determine the age of the sediments from the high terrace of the Somme River at Abbeville. Electron Spin Resonance (ESR) dating was applied to quartz grains extracted from the sediments from Moulin Quignon (Fig. 4). The Electron Spin Resonance (ESR) dating method is based on the accumulation during time of paramagnetic electrons in mineral defects resulting from environmental natural irradiation (e.g.^46^). ESR dating of quartz is based on the resetting of the signal by sunlight exposure at the time of deposition^47^. An ESR age is derived by assessing the total dose received by the sample since its deposition divided by the dose rate which is obtained from all environmental radiation sources including internal sources and cosmic rays.

In this work, the ESR signal of the Aluminium (Al) centre was used. The preparation protocol followed for quartz grain extraction is from^48^. After quartz extraction, each sample was split in eleven aliquots. Nine of them were irradiated, respectively at 268, 412, 659, 976, 1580, 2550, 4200, 7340 and 10300 Gy, with a gamma 60Co source (CEA Saclay, France) emitting 1.25 MeV radiation with a dose rate of about 200 Gy/h; the tenth aliquot was conserved as a reference for the geological (natural) irradiation and the eleventh aliquot was exposed during 1000 h to light in a Dr Honhle SOL2 solar simulator (light intensity between 3.2 and 3.4 105 Lux) in order to determine the unbleachable part of the ESR-Al signal.

Each set of aliquots was measured at least three times by ESR using a Bruker EMX spectrometer cooled by liquid nitrogen at 107 K and each aliquot was measured three times each after an approximately 120° rotation of the tube in the ESR cavity, in order to consider the angular dependence of the signal due to sample heterogeneity. The following ESR acquisition parameters were used: 5 mW microwave power, 1024 points resolution, 20 mT sweep width, 100 kHz modulation frequency, 0.1 mT modulation amplitude, 40 ms conversion time, 20 ms time constant and 1 scan. The signal intensity of Al-centre was measured between the top of the first peak at g = 2.018 and the bottom of the 16th peak at g = 2.002 of the Aluminium hyperfine structure^49^. Equivalent doses (D_e_) were determined from the obtained ESR intensities vs dose curve using an exponential + linear function^50^ using Microcal OriginPro 8 software with 1/I² weighting.

In the age calculation, the dose rates d_a_ were calculated from the radionuclide contents U, Th, K) of the sediments taking into account *in situ* gamma-ray measurements performed with an Inspector 1000 (Canberra) gamma spectrometer and the location of the samples in the stratigraphic sequence. Water content (%) was estimated by the difference in mass between the natural sample and the same sample dried for a week in an oven at 50 °C and water attenuation values from Grün^51^. Dose rates were determined taking into account alpha and beta attenuations estimated for the selected grain sizes from the tables of Brennan^52,53^; Dose rate conversion factors from Guérin *et al*.^54^ k-value of 0.15^47^ The internal dose rate was considered as negligible due to the low content of radionuclides from the quartz grains^55,56^. We removed the external part of the grain (around 20 µm) by HF etching; cosmic dose rate calculated from the equations of^57^ corrected according to the altitude and latitude. The bleaching rate δ_bl_ (%) is determined by comparison of the ESR intensities of the natural and bleached aliquots (δ_bl_ = ((I_nat_ − I_bl_)/I_nat_) × 100). ESR age estimates are given with one sigma error range^6^.

Radiometric data from the three Moulin Quignon sediment samples (MQ17-01, MQ17-03 and MQ 17-05), obtained by laboratory gamma measurements (analytical uncertainties given with ±1σ), allow the determination of alpha and beta rays contributions to the annual dose (Table S1).

## Supplementary information

Supplementary info

## Data Availability

All data generated or analysed during this study are included in this published article (and its Supplementary Information files).
