# Supplementary material for: The earliest evidence of Acheulian occupation in Northwest Europe and the rediscovery of the Moulin Quignon site, Somme valley, France
Source: Sci Rep. 2019 Sep 11;9:13091. doi: 10.1038/s41598-019-49400-w (PMC6739401; doi:10.1038/s41598-019-49400-w)
Supplement: Supplementary file 1 — Supplementary info [file 41598_2019_49400_MOESM1_ESM.pdf]

Supplementary information

The earliest evidence of Acheulian occupation in Northwest Europe and the rediscovery of the *Moulin Quignon* site, Somme valley, France

Pierre ANTOINE [(a*)], Marie-Hélène MONCEL[(b)], Pierre VOINCHET [(b)], Jean-Luc LOCHT [(c,a)], Daniel AMSELEM [(b)], David HERISSON [(d)], Arnaud HUREL[(b)] , Jean-Jacques BAHAIN [(b)].

(a*) Laboratoire de Géographie Physique, Environnements Quaternaires et actuels, 1 Place A. Briand, 92195 Meudon Cedex, France.

(b) UMR 7194 Histoire naturelle de l'Homme préhistorique (MNHN- CNRS-UPVD), 1 rue René Panhard, 75013 Paris France.

(c) Institut National de la Recherche archéologique préventive (INRAP) Nord-Picardie, 32 avenue de l'Etoile du Sud, 80440 Glisy, France.

(d) UMR 7041 CNRS-Univ. Paris X Nanterre, Maison Archéologie & Ethnologie, René-Ginouvès, 21, allée de l'Université, 92023, Nanterre Cedex, France.

a* Corresponding author : pierre.antoine@lgp.cnrs.fr.

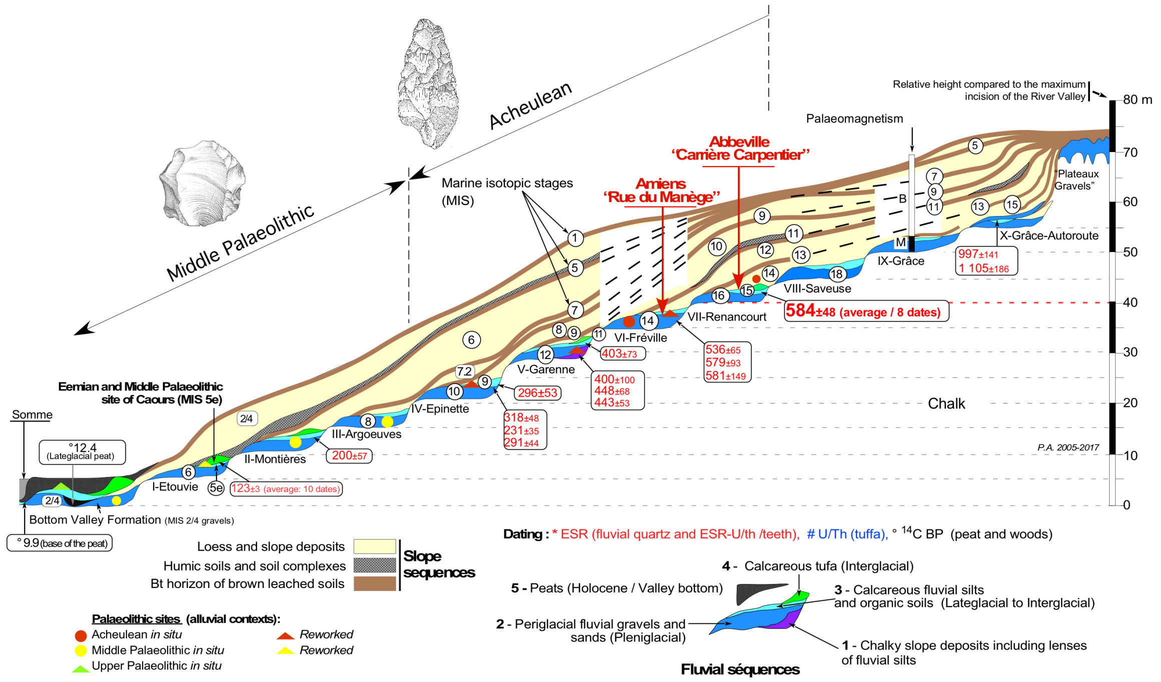

Figure S1. The Middle Somme valley Quaternary terrace system: morpho-stratigraphy, dating and correlations with MIS stages, according to (24, 25). In the whole system, alluvial formations are composed by a fluvial sequence including from the base to the top the following succession of four main phases allocated to a single glacial-interglacial cycle:

1) Early-glacial: heterogeneous coarse sediments made up of slightly reworked large chalk blocks and flint nodules embedded in a calcareous sandy matrix, including some thin lenses of fluvial calcareous silts (slope deposits slightly reworked by fluvial processes). These deposits are only preserved in the outcrops located very close to the former slope of the valley, as in the Garenne or Carpentier sequences. They are globally allocated to continental temperate conditions.

2) *Full glacial*: thick body of well-sorted flint gravels including a few sand lenses (braided river system) forming the main sedimentary body of the sequence (3 to 4 m in thickness). The base of this unit frequently exhibits large Tertiary sandstone blocks reworked by periglacial processes from the slopes to the alluvial plain (very cold conditions, open landscape and periglacial environment, full glacial).

3) *Lateglacial to early interglacial*: calcareous sandy silts, more or less laminated (0.5 to 1.5m in thickness), characterized by temperate faunas (molluscs, mammals) to the top. This unit, covering the alluvial sequence, is frequently overlaid by thin organic grey marshy soil horizons (≈ 0.1m).

4) *Interglacial optimum*: in a few sequences, calcareous tufa beds (0,5 to 3.5 m in thickness), characterised by fully temperate bio-indicators, and especially mollusc assemblages, record the interglacial optimum as in Caours (Eemian / MIS 5e) or Saint-Acheul (MIS 11).

		Upper Middle Somme (Amiens system)	Lower Middle Somme (Abbeville system)		
RH (m)	Number	Alluvial Formation	Alluvial Formation	fluvial sequences	MIS
**+ 5-6m**	**I**	***Etouvie Fm***	***Port-le-Grand Fm***		**5e 6**
+ 10-12	II	*Montières Fm*	*?*		7.1 7.2
**+ 14-15**	**III**	***Argoeuves Fm***	***Cambron-Menchecourt Fm***		**7.3 8**
+ 20-21	IV	*Epinette Fm*	*Mautort Fm*		9 10
**+ 27-29**	**V**	***Garenne Fm***	***Champ de Mars Fm.?***		**12**
+ 35	VI	*Fréville Fm*	*Mareuil Fm*		13 14
**+ 40**	**VII**	***Renancourt Fm***	***Carrière Carpentier Fm***	***MB***	**15 16**
+ 44-45	VIII	*Saveuse Fm*	*Mareuil-Caubert Fm*		17 18
**+ 50-52**	**IX**	***Grâce Fm***	***Caubert Fm***		**19 20**
+ 55	X	*Grâce-Autoroute Fm*	*?*		≤ 21
**+ 59-60**	**XI**	***Chaîne d'Or***	**?**		
+ 70 / 75		Plateau gravels	?		

1 2 3 4 5 ***MB**: Marne Blanche / White Marl*

Figure S2. The stepped terrace systems of the Somme basin and associated sedimentary sequences; reference systems at Amiens and Abbeville and correlation with MIS succession for the last 1 Ma according to (24).

1) Heterogeneous coarse gravels with unrolled flint and chalk blocks, calcareous silty matrix and lenses of calcareous fluvial silts (Early-glacial part of the cycle).

2) Coarse flint gravels (flint and chalk) with calcareous sandy matrix, sandy lenses and some ice-rafted Tertiary sandstone blocks (full glacial)

3) Calcareous silts and sandy silts deposited as overbank deposits in an alluvial plain connected to a single channel meandering river (beginning of the Interglacial period)

4) Organic marshy soils and tufa deposits: Interglacial optimum.

5) Alluvial Formation X only: stratified coarse fluvial sands (end of the Interglacial).

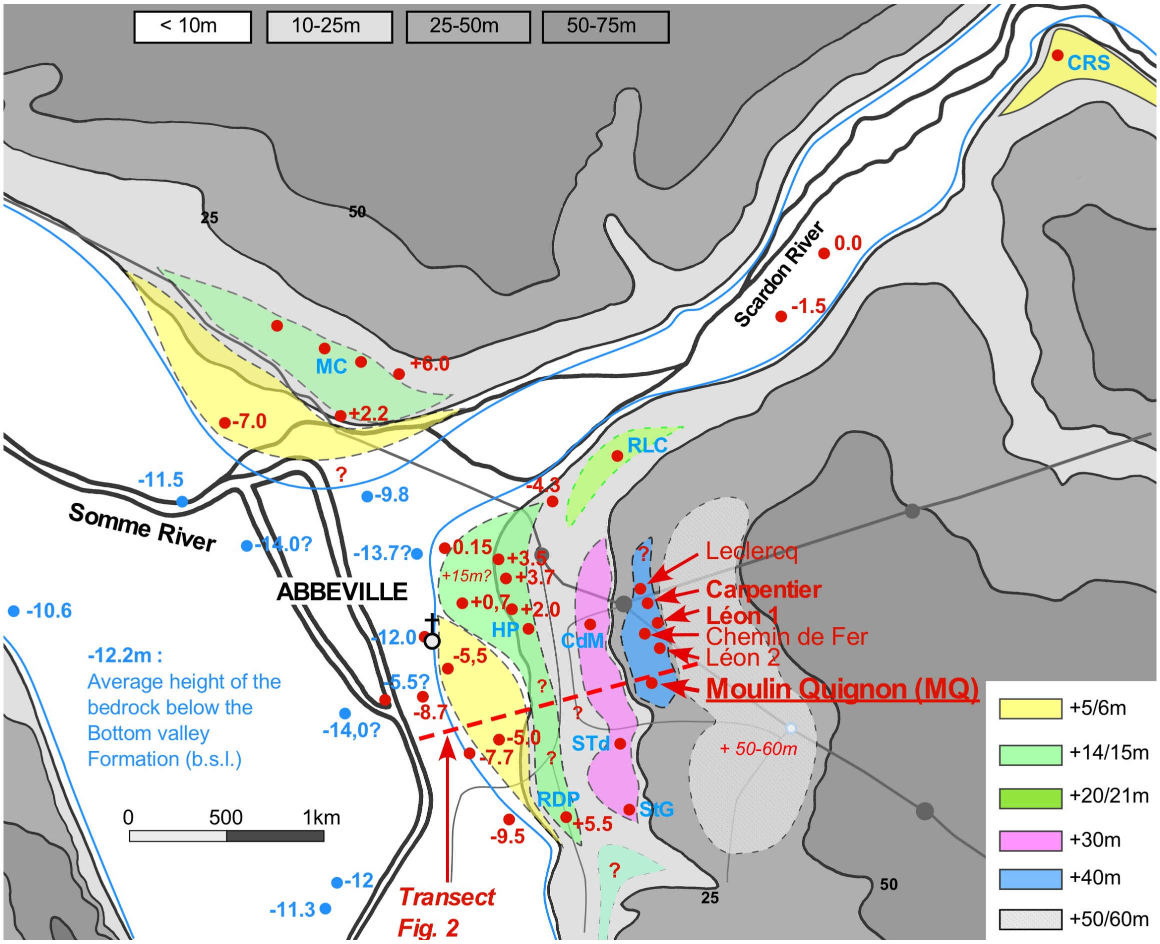

Figure S3. Location of the Moulin Quignon, Léon 1&2, Chemin de Fer, Carpentier and Leclercq sites on a map of the Quaternary alluvial formations of the Abbeville area according to (25), modified.

Red dotted line : transect of Fig. 2A. Other sites: RdP: Rue de Paris, Std: Abbeville-Stade, CdM: Abbeville-Champ de Mars, HP: Abbeville-Hôpital, MC: Abbeville-Menchecourt, RLC: Abbeville-Rue du Lieutenant Caron, CRS: Caours.

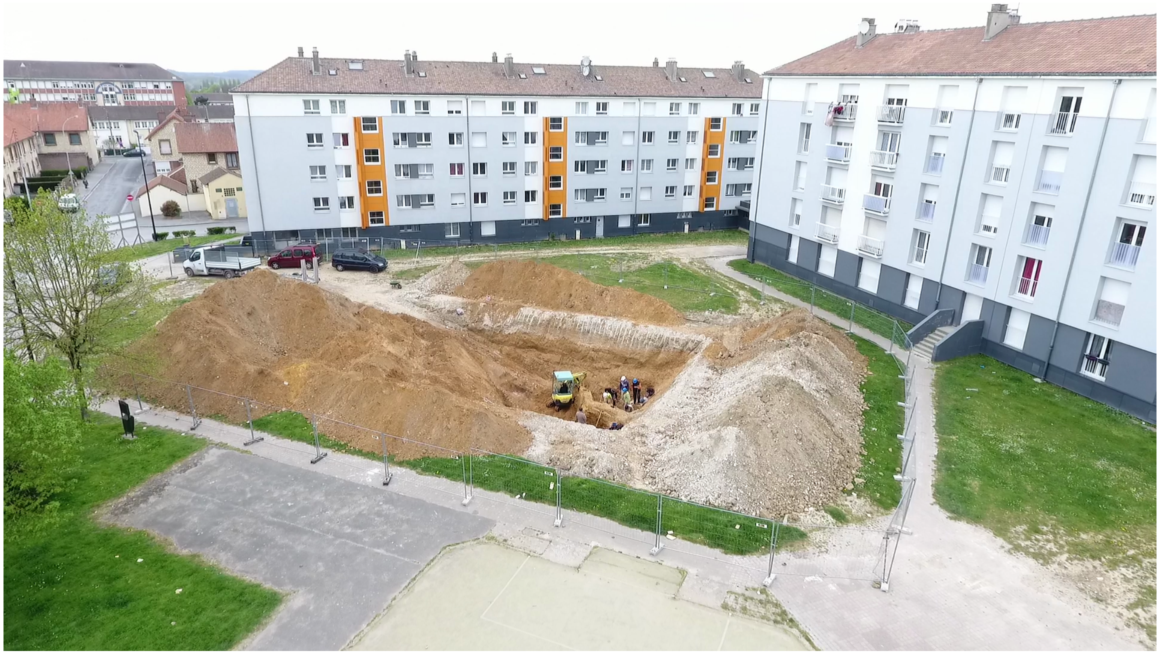

Figure S4. Aerial view of the archaeological excavation opened at Abbeville between the buildings on the location of the former Moulin Quignon site (April 2017, drone photograph by L. Lapo).

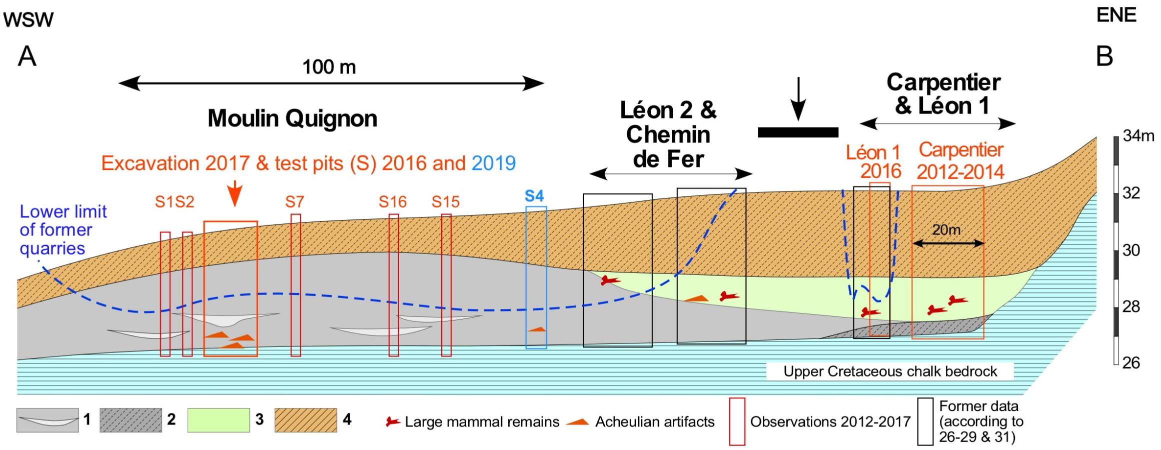

Figure S5. Cross-section of the main units of the Alluvial Formation (1-3) and of the slope deposits (4) of the Carpentier Formation between Moulin Quignon and the Carpentier quarry. This profile (real altitudes and thicknesses) has been constructed by the projection of all the stratigraphic information obtained from recent research in Carpentier (25), Léon 1 and Moulin Quignon sites (2016-2019) and former descriptions (26-29) on one transect (240m) perpendicular to the main axis of the alluvial formation (see Fig. 3 for captions).

1) Poorly sorted and heterometric flint gravels including numerous unrolled flint nodules and chalk blocks packed in a calcareous sandy matrix.

2) Periglacial fluvial gravels and sand lenses.

3) *White marl* complex: interglacial calcareous silts and sandy silts with oncolith sand layers and large mammal remains (Cromerian III / MIS 15).

4) Undifferentiated slope deposits: reworked clayey gravels, sand layers, sandy loess and palaeosols.

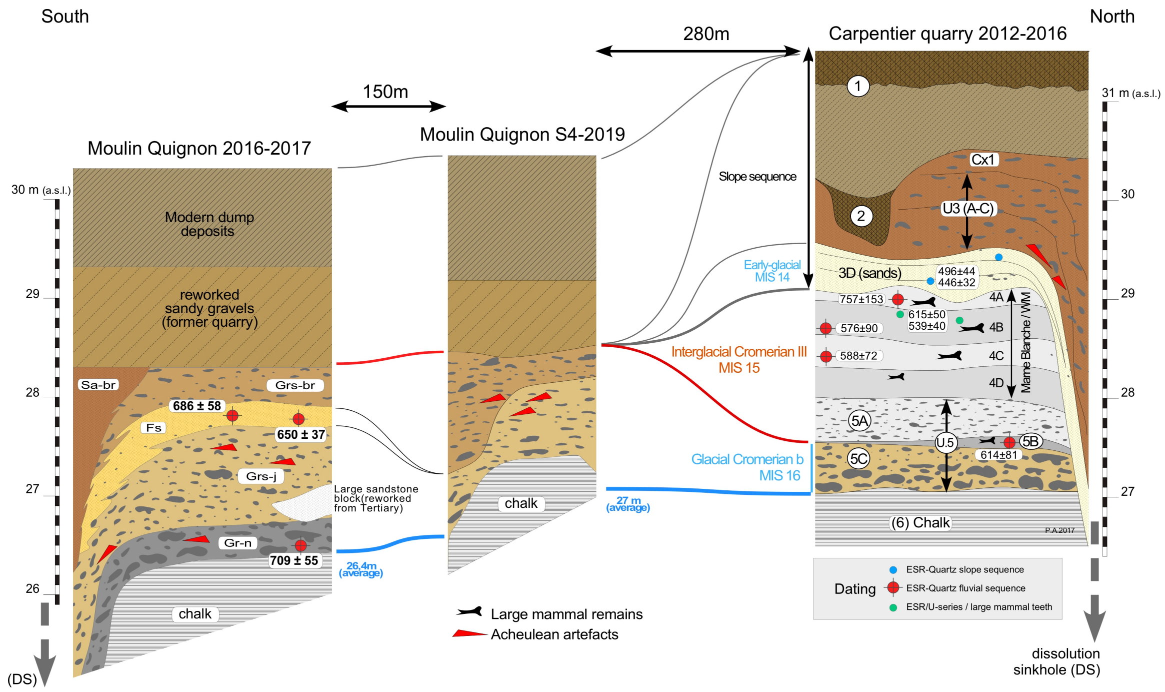

Figure S6. Detailed correlations between the Moulin Quignon and Carrière Carpentier sequences. Description of units from Carrière Carpentier and ESR dating according to (25). Units from Moulin Quignon are described in the caption of Fig. 4.

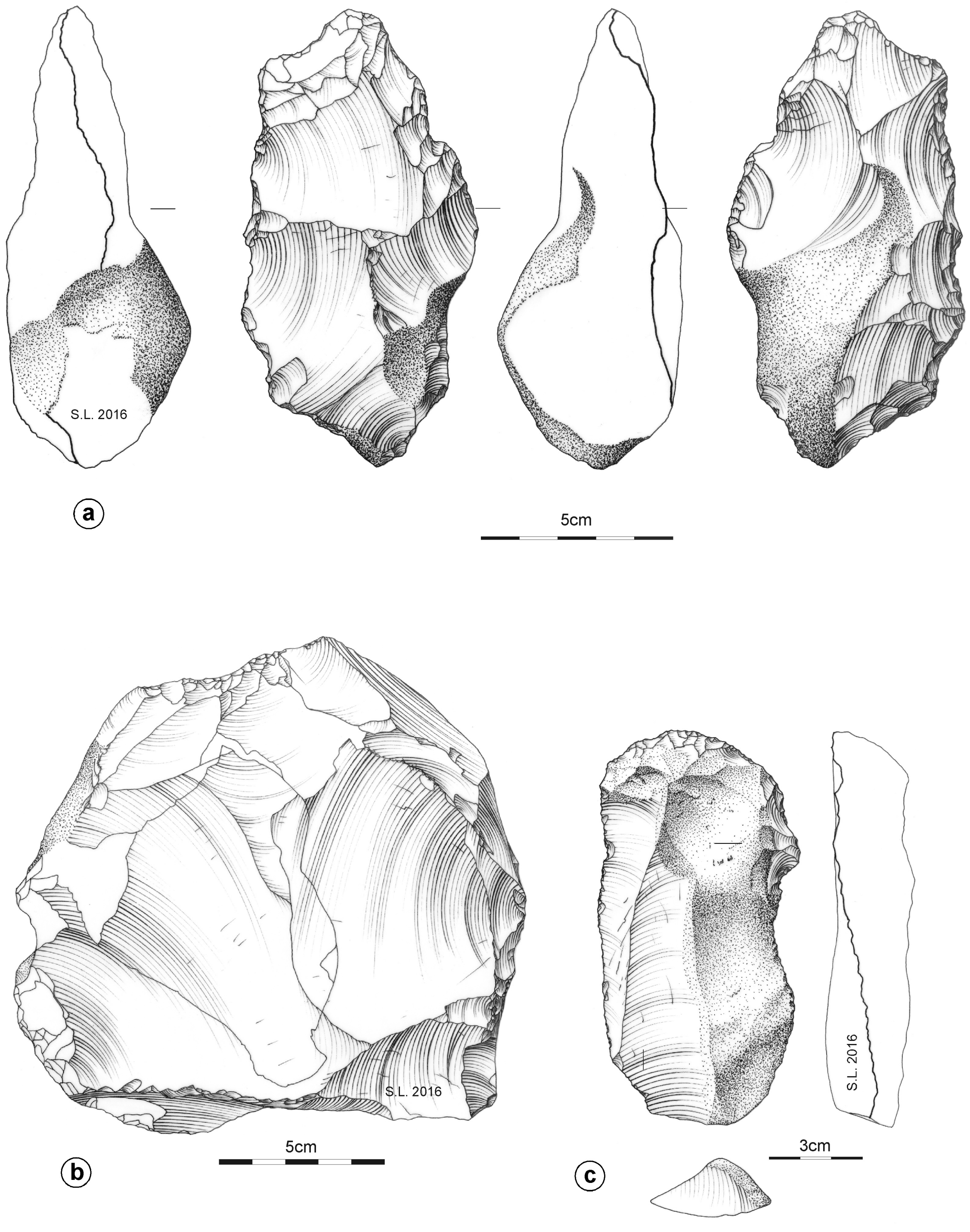

Figure S7. Palaeolithic artefacts from Moulin Quignon (drawings by S. Lancelot, INRAP). (**a**) Biface from the lower part of test pit S.12-2016 (base of the lower gravels "black layer", Gr-n), (**b**) Large nucleus from test-pit S.12.12-2016 (Lower sandy gravels, Grs-j). (**c**) Scraper on a thick laminar flake recovered in test-pit S.18 (Lower sandy gravels, Grs-j).

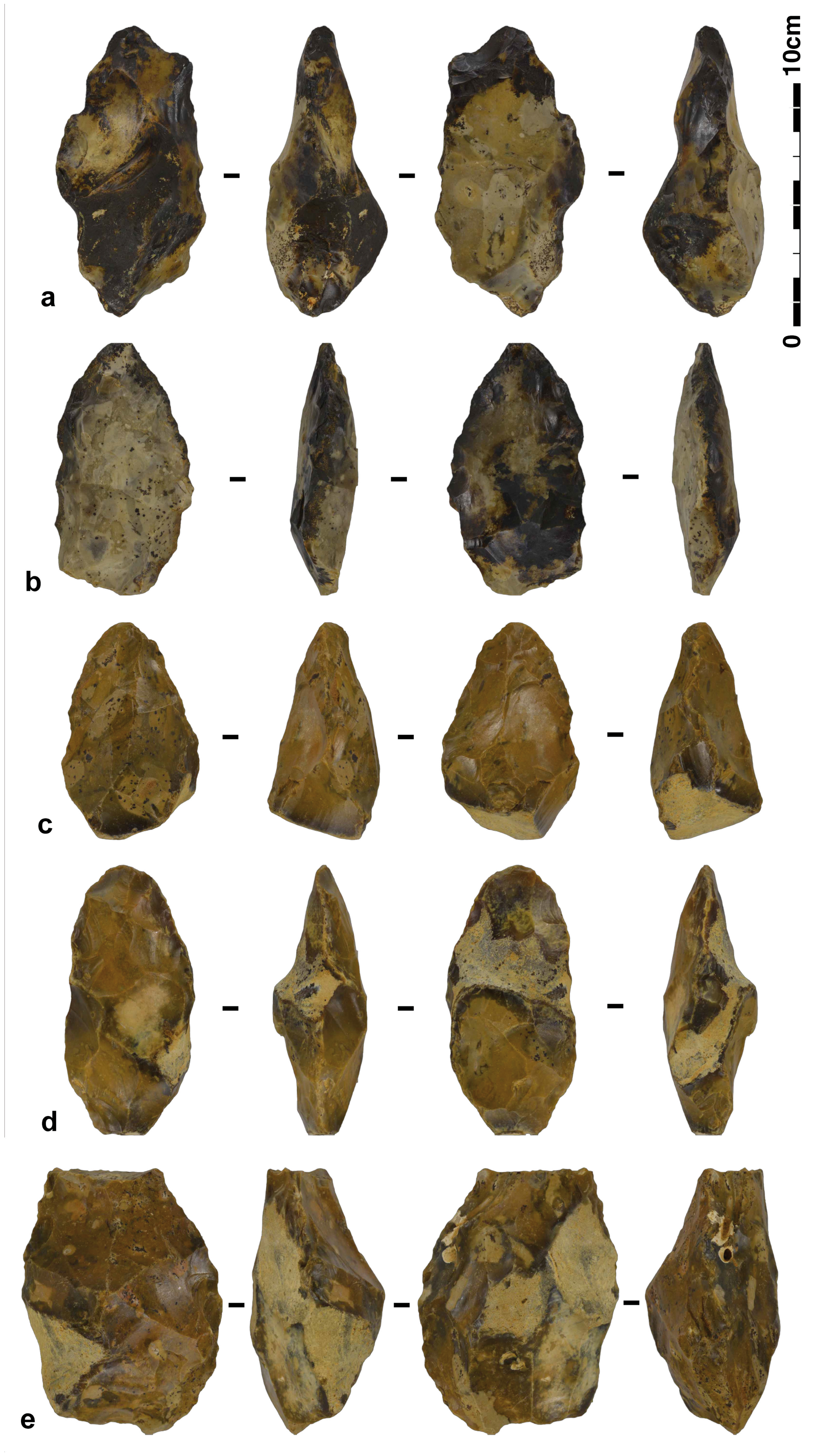

Figure S8. Ortho-photographs extracted from 3D models of the five bifaces discovered at Moulin Quignon during the test pits (a) and excavations (b, c, d, e) (photographs and 3D models by D. H.). a) crudely-shaped biface, b) pointed biface without cortex, c) pointed biface with a thick cortical base, d) oval biface with cortical patches, e) asymmetrical biface with broken tip.

Table S1. Annual dose rate contributors of the Moulin Quignon sediments - radioelement activities, water content (w), sampling depth, contributions of different radiations.

**Samples**	U (ppm)	Th (ppm)	K (%)	W (%)	depth (cm)	D_{Cosm} (µGy/a)	D_{α} (µGy/a)	D_{β} (µGy/a)	D_{γ} (µGy/a)
**MQ17-01**	0,200±0,057	0,817±0,078	0,227±0,008	10	360	109 ± 5	6±1	188±13	201 ± 11
**MQ17-03**	0,538±0,066	1,746±0,096	0,336±0,010	10	245	131 ± 7	15±1	316±16	199 ± 13
**MQ17-05**	0,261±0,051	0,643±0,064	0,124±0,006	10	380	106 ± 5	6±1	124±11	110 ± 9
